# Our New York Letter

**Published:** 1890-04

**Authors:** C. D. Roy

**Affiliations:** Charity Hospital; New York


### Correspondence.

NEW YORK, March 5, 1890.

The temperature which has prevailed in New York this winter reminds me much more of the seasons in Atlanta than the ones which are generally experienced in this latitude. The continuous rainy and muddy weather which usually prevails here in the spring has not yet come upon us, although there has been no paucity of showers. The various medical colleges in the city are now busily engaged in their preparations for closing the session's work. The graduating exercises will send forth innumerable doctors, and the South will be able to claim no small proportion of that number. The epidemic of influenza seems to have subsided, judging from the decrease in the number of bronchial cases admitted into the hospital. Every patient admitted into that institution is provided with a card upon which is given the name in full, age, civil condition, nativity and occupation. By this method correct statistics may be kept, and any other information which may be desired. Patients are first carried into a reception room, where they are vaccinated by the junior assistant, and then distributed to the various wards by the house physician in charge. Before entering a ward each patient is required to take a thorough bath, which often immediately improves his physical condition. All the wards are kept continously full, and oftimes patients have to be returned to the city for want of space. A comparatively recent treatment of orchitis and epidymitis is now much in vogue at the hospital, and a method which has given most excellent results. The plan is a simple one, and one which accomplishes the result often aimed at with more radical means. In an acute orchitis or epididymitis there is an engorgement of the blood vessels, with more or less transudation of the inflammatory products, thus causing the increased pressure on the terminal ends and trunks of the nerves in the testicle. This organ itself is red hot, swollen and very painful to the touch, presenting all the symptoms which it is, of an acute inflammation. Now, what are the rational signs for treatment? In the first place, and that which most interests the patient, we desire to ameliorate the pain. In the treatment of any inflammation, be it superficial or deep and extensive, it requires for its successful management that it should be treated on rational grounds, and in no blind haphazard manner. Whether the iflammation was caused sympathetically by metastasis or by the direct absorption of the virus does not so much immediately concern us as does the removal of the present irritative symptoms. What now is the cause of the pain experienced in the inflammation in question? As we have said, it is the abnormal afflux of blood to the part, the transudation of inflammatory products, thus causing increased pressure on the nerves. Hence, to remove the pain, the increased pressure must be removed. By accomplishing the latter result you also remove the other symptoms of heat, redness and swelling. The results aimed at have been accomplished, in a great measure, by some of the older methods of treatment. For instance, puncture and scarification have been resorted to with good results, their mode of action being by letting out directly the engorging material. The application of ice to the scrotum is one of the most frequent remedies resorted to, which, by its constringing effect on the blood vessels, lessens the amount of blood to the part, thus lessening the engorgement, and at the same time producing an anæsthetic state of the nerves. The method in question accomplishes these results by causing the watery portions of the blood and products of inflammation to be eliminated by means of the sudoriferous ducts. Not only by the sweat ducts do the products escape, but there seems to be an actual transudation from the blood vessels themselves. An analogous example is afforded in systemic fever, when we aid in decreasing the temperature by increasing the heat-loss through the means of bodily sweat. The method is as follows: A piece of absorbent cotton, from a quarter to half an inch in thickness, is cut in the shape of a triangle. This triangular piece is then placed around the enlarged testicle, in such a manner that the base, being at the lower portion of the scrotum, the three angles are brought up and gathered around the scrotum at the base of the penis. Around this, on the outside is placed a layer of oiled silk, and all this lightly covered with a securing bandage. The scrotum, in the meantime, is kept supported, and it is best for the patient to remain in bed, if possible. In about two days the inflammatory symptoms will almost have subsided, and in a week recovery may be declared. The method seems so simple that one may be almost incredulous, but the results obtained are so excellent as to warrant my describing the method to the readers of the RECORD, and thus give them an opportunity of testing its value. Being connected with the service upon which Dr. F. R. Sturgis was the visiting surgeon, I was somewhat interested to hear of the different ways in which different distiguished surgeons treated strictures of the urethra. Dr. S. is now almost the only man of note who accasionally treats them by the method of divulsion. He has an instrument of his own, made after the order of a glove-stretcher. This method he, of course, resorts to in strictures of large calibre. Having regulated the divulsor to the size he wishes to divulge the stricture, he introduces the instrument closed, and after having passed the stricture, he opens the blades several times, and finally withdraws them in the separated condition. I cannot say that I favor the method in question, for the cases upon whom it was tried did not give the best results. Dr. Phelps has devised an ingenious little instrument to be used in circumcision, by which that operation is made much easier, quicker and neater. It is nothing more than a pair of forceps, with its blades crossed in such a manner that when one extremity is pressed the other opens, the latter being curved out, and pointed at its extremity in such a manner as to grasp the mucous membrane when inserted within the prepuce, and extended. The instrument is placed just within the full edge, in such a manner that when the blades are separated the mucous membrane is seized by the pointed extremities, and thus both it and the integument are pulled forward at the same time. A dorsal slit is then made with the scissors, while the prepuce is thus extended in the horizontal position, with the instrument, and then the incision is completed on the sides, so as to sever the fraenum near the free border of the prepuce. By this method you cut the integument and mucous membrane at the same time, and thus avoid a second cutting on the latter. The quikeness by which this method aids you in doing the operation makes it of no little value. C. D. ROY, M. D.

*Charity Hospital.*

